# Supplementary material for: Gaussian graphical modeling of the serum exposome and metabolome reveals interactions between environmental chemicals and endogenous metabolites
Source: Sci Rep. 2021 Apr 7;11:7607. doi: 10.1038/s41598-021-87070-9 (PMC8027000; doi:10.1038/s41598-021-87070-9)
Supplement: Supplementary file 1 — Supplementary Information [file 41598_2021_87070_MOESM1_ESM.docx]

**Supplementary Information**

Gaussian graphical modeling of the serum exposome and metabolome reveals interactions between environmental chemicals and endogenous metabolites

Vincent Bessonneau, Roy R. Gerona, Jessica Trowbridge, Rachel Grashow, Thomas Lin**,** Heather Buren, Rachel Morello-Frosch^*^, Ruthann A. Rudel^*^

**Methods**

**Metabolomics data processing**

Metabolomics features were annotated using the R package “xMSannotator”^1^. The xMSannotator package is an integrative scoring algorithm that integrates i) correlation analysis among all measured m/z features, ii) data-driven network of modularity analysis to find modules of co-expressing m/z features, iii) retention-time based clustering to find co-eluting m/z features, iv) mass defect analysis to identify group of features that follow an isotopic pattern or potential adducts or in-source fragmentation, and v) matching m/z features against LC-HRMS libraries.

Data pre-processing using the R-package XCMS^2^

####R-script for pre-processing LC-QTOF/MS metabolomics data using XCMS

library(xcms)

mzxml_files<-"C:/LC_QTOF_data_files" #directory that contain LC-QTOF/MS raw data files

##Peak picking using "centwave" from centroid LC-QTOF/MS data ###

xset<-xcmsSet(mzxml_files, ppm=15,method="centWave",

peakwidth=c(5,20),mzdiff=0.015,prefilter=c(3,5000),snthresh=6)

##Grouping peaks detected in at least 50% of individuals/group

xset1<-group(xset,bw=5,minfrac=0.5,mzwid=0.015,max=100,minsamp=1)

##Retention time correction

xset2<-retcor(xset1,method="obiwarp",plottype=c("deviation"))

####Grouping peaks detected in at least 2 individuals/group - 3% min fraction

xset3<-group(xset2,bw=5,minfrac=0.5,mzwid=0.015,max=100,minsamp=1)

##Fill in peaks

xset4<-fillPeaks(xset3)

##Create report

report<-diffreport(xset4,"FF","OW","xcms_preprocess",10)

Annotation of metabolomics features using the R-package XMSannotator^1^

####R script for annotation of LC-QTOF/MS metabolomics features

library(xMSannotator)

dataA<-xcms_preprocess.csv #data file (.csv) that contains m/z and intensity of metabolomics features processed with XCMS

outloc<-"C:\Users...." #Set up output location

max.mz.diff<-10 #mass search tolerance for DB matching in ppm

max.rt.diff<-10 #retention time tolerance between adducts/isotopes

corthresh<-0.7 #correlation threshold between adducts/isotopes

max_isp=5

mass_defect_window=0.01

num_nodes<-2 #number of cores to be used; 2 is recommended for desktop computers due to high memory consumption

db_name="HMDB"

status="Detected and Quantified"

num_sets<-300 #number of sets into which the total number of database entries should be split into;

mode<-"neg" #ionization mode

queryadductlist=c("M-H","M-H2O-H","M+Na-2H","M+Cl","M+FA-H")

adduct_weights<-NA

customIDs<-NA

#########################

dataA<-unique(dataA)

print(dim(dataA))

system.time(annotres<-multilevelannotation(dataA=dataA,max.mz.diff=max.mz.diff,max.rt.diff=max.rt.diff,cormethod="pearson",num_nodes=num_nodes,queryadductlist=queryadductlist, mode=mode,outloc=outloc,db_name=db_name, adduct_weights=adduct_weights,num_sets=num_sets,allsteps=TRUE, corthresh=corthresh,NOPS_check=TRUE,customIDs=customIDs,missing.value=NA,deepsplit=2,networktype="unsigned", minclustsize=10,module.merge.dissimilarity=0.2,filter.by=c("M-H"),biofluid.location=NA,origin=NA,status=status,boostIDs=NA,max_isp=max_isp, HMDBselect="union",mass_defect_window=mass_defect_window,pathwaycheckmode="pm",mass_defect_mode="pos"))

Computing Gaussian Graphical Models of the serum chemical exposome with the metabolome using the R-package GeneNet^3^

##R script for computing Gaussian Graphical Models (GGMs) of the serum exposome and metabolome

library(GeneNet)

#Data matrix containing *m/z* intensities of environmental chemicals and endogenous metabolites:

#for the whole cohort, the data matrix contains [143 participants x (52 environmental chemicals + 90 endogenous)]

data.x<-as.matrix(data[,2:145])

#Compute GGMs

pcor.dyn<-ggm.estimate.pcor(data.x,method="dynamic")

#estimating optimal shrinkage

met.edges<-network.test.edges(pcor.dyn,direct=TRUE)

#Extract network containing significant association (i.e edges) with probability>0.9 (i.e. FDR<0.1)

net<-extract.network(met.edges, cutoff.ggm = 0.9)

**NHANES exposures, covariates and outcome measurements**

Perfluoroalkyl substances (PFAS) concentrations

PFASs were measured in serum by the National Center for Environmental Health (NCEH) using automated solid-phase extraction coupled to reversed-phase high-performance liquid chromatography-tandem mass spectrometry, as described elsewhere^4^. Our study examined pefluorohexane sulfonic acid (PFHxS) and perfluorooctane sulfonic acid (PFOS). Values below the limit of detection (LOD) were reported by NHANES as the LOD divided by the square root of 2.

Parabens concentrations

Parabens were measured in urine samples using online solid-phase extraction coupled to reversed-phase high-performance liquid chromatography-tandem mass spectrometry, as described elsewhere^5^. Our study examined ethyl paraben (EPB) and butyl paraben (BPB). Values below the limit of detection (LOD) were reported by NHANES as the LOD divided by the square root of 2.

Metabolic syndrome

To define metabolic syndrome (MetS), we used data on waist circumference, blood pressure, antihypertensive and anti-diabetic medication, and serum measurements of triglycerides, high density lipid (HDL) cholesterol, and fasting blood glucose. Data on waist circumference and blood pressure were collected as described in the NHANES Anthropometry Procedures Manual^6^. Fasting blood glucose, HDL cholesterol, and triglyceride levels were measured using methods described in the NHANES Laboratory/Medical Technologists Procedures Manual^7^. Information on antihypertensive and anti-diabetic medication were self-reported and collected from study participants.

C-reactive protein (CRP) concentrations and complete blood count

CRP levels were measured in serum by latex-enhanced nephelometry, as described elsewhere^8^. The LOD was 0.01 mg/dL. Complete blood count (CBC) was based on the Beckman Coulter method of counting and sizing white blood cells, lymphocytes, neutrophils, monocytes, eosinophils, basophils and platelets in whole blood, as described in the NHANES Laboratory Procedures Manual^7^.

Covariates

Age, sex, race/ethnicity, physical activity, smoking status, poverty status, and total caloric intake in women were self-reported by questionnaire. Body mass index (BMI) was derived from examination measurements. Serum cotinine – the major metabolite of nicotine - was measured using solid-phase extraction and reversed-phase high-performance liquid chromatography-tandem mass spectrometry, as described in the NHANES Laboratory Procedures Manual^7^. Values below the limit of detection (LOD) were reported by NHANES as the LOD divided by the square root of 2.

For chemicals associated with bile acids in GGM networks, ORs and association estimates were also reported before and after further adjustment for use of antibiotics in the past 30 days since the gut microbiota is a possible confounder of associations between exposures to these chemicals and outcome variables related to MetS and inflammation. For each NHANES cycle, data on prescription medications used in the past 30 days, including drug name and duration of use, were collected through direct abstraction from prescription medication containers during the household interview as described in the NHANES Dietary Supplements and Prescription Medication Questionnaire^9^. The drug names were converted into a standard generic drug name and a therapeutic drug class was assigned based on the Multum Lexicon Drug Database^10^. To generate a binary variable indicating that a participant had taken an antibiotic in the past 30 days, we selected only drugs within the first-level category, “anti-invectives”. Topical antibiotics were also excluded from the analysis.

**Confirmation of chemical exposures measured using non-targeted LC-QTOF/MS in serum samples from women firefighters and office workers**

We confirmed the presence of annotated environmental chemicals in the serum samples by running the LC-QTOF/MS analysis using the corresponding reference standard spiked into synthetic serum. Annotated chemicals from participants samples were confirmed if the *m/z* (± 10 ppm), at least two fragment peaks in the MS/MS spectra, and the relative retention time (RRT) (± 0.1 min) of the authentic standard matched those found in the serum samples, consistent with level 1 confidence in identification^11^. The RRT was calculated by dividing the RT of each analyte by the RT of an internal standard spiked in serum samples before sample preparation (mono-(2-ethylhexyl)-phthalate-C13).

**Table S1. Summary of relative retention time and exact mass of chemical exposures measured using LC-QTOF/MS (ESI-) in women firefighters and office workers and identified as level 1 according to the Metabolomics Standard Initiative**

|  |  | **Experimental data** | | **Validation data** | |
| --- | --- | --- | --- | --- | --- |
| **Chemical Name** | **Chemical Class** | **RRT^1^ (min)** | **Exact mass^2^** | **RRT^1^ (min)** | **Exact mass^2^** |
| PFHxS | PFAS | 1.03 | 399.9441 | 1.02 | 399.9443 |
| PFOS | PFAS | 1.12 | 499.9378 | 1.09 | 499.9380 |
| Ethyl paraben | Phenol | 0.92 | 166.0631 | 0.93 | 166.0633 |
| Butyl paraben | Phenol | 1.03 | 194.0943 | 1.06 | 194.0946 |
| Pentachlorophenol | Phenol | 1.01 | 263.8471 | 1.09 | 263.8474 |
| Eugenol | Phenol | 1.03 | 178.0994 | 1.07 | NA |
| Mono-(2-ethylhexyl)-phthalate (MEHP) | Phthalate | 0.98 | 278.1518 | 0.97 | 278.1519 |
| Diphenyl phosphate | Phosphate flame retardant | 0.78 | 250.0396 | 0.79 | 250.0391 |

^1^Relative retention time (RRT)

^2^Calculated exact mass for the neutral molecule based on the measured *m/z*

**Results**

**Table S2**. **Summary of significant partial correlations between compounds found in women firefighters and office workers**

| **Compound 1 (ID level^1^)** | **Compound 2 (ID level^1^)** | **PCC^2^** | ***P* value** |
| --- | --- | --- | --- |
| 2-(1,1-Dimethylethyl)phenol (3) | Thymol (3) | 0.49 | 2.22E-16 |
| Caprylic acid (3) | Capric acid (2) | 0.43 | 2.22E-16 |
| p-Cresol (3) | p-Cresol sulfate (2) | 0.37 | 2.22E-16 |
| Glycocholic acid (3) | Cholesterol sulfate (3) | 0.35 | 2.22E-16 |
| Androsterone glucuronide (2) | 11-beta-Hydroxyandrosterone-3-glucuronide (2) | 0.33 | 2.22E-15 |
| Dodecanoic acid (2) | 6-Tetradecenoic acid (2) | 0.33 | 5.33E-15 |
| Myristic acid (2) | 12(13)Ep-9-KODE (2) | 0.32 | 1.02E-14 |
| 2-Methoxyestrone (3) | 19-Hydroxyandrost-4-ene-3,17-dione (2) | 0.32 | 2.44E-14 |
| 2-Arachidonylglycerol (3) | Chenodeoxycholic acid (3) | 0.29 | 1.82E-12 |
| Lithocholic acid (3) | Sulfolithocholylglycine (2) | 0.28 | 1.70E-11 |
| Cholesterol sulfate (3) | Taurocholic acid (3) | 0.28 | 3.44E-11 |
| Taurodeoxycholic acid (2) | Taurocholic acid (3) | 0.28 | 4.94E-11 |
| Eicosapentaenoic acid (2) | Docosahexaenoic acid (2) | 0.26 | 5.09E-10 |
| LysoPE(18:0) (2) | APGPR Enterostatin (3) | 0.25 | 1.55E-09 |
| Glycocholic acid (3) | Taurocholic acid (3) | 0.25 | 2.07E-09 |
| Indoxyl sulfate (2) | Alpha-N-Phenylacetyl-L-glutamine (3) | 0.25 | 2.61E-09 |
| Arachidonic acid (3) | 15S-HETE (3) | 0.25 | 3.72E-09 |
| LysoPC(14:0) (2) | LysoPC(16:0) (2) | 0.25 | 4.27E-09 |
| 15S-HETE (3) | 19(20)-EpDPE (2) | 0.25 | 4.29E-09 |
| Pregnenolone sulfate (3) | 17-Hydroxypregnenolone sulfate (2) | 0.25 | 4.72E-09 |
| 5-KETE (2) | 19(20)-EpDPE (2) | 0.24 | 8.13E-09 |
| Lithocholic acid (3) | Lithocholic acid glycine conjugate (3) | 0.23 | 3.34E-08 |
| Methyl eugenol (3) | Butyl paraben (1) | 0.23 | 5.92E-08 |
| Monoisotridecyl phthalate (3) | 5a-Tetrahydrocorticosterone (3) | 0.22 | 1.29E-07 |
| Indoxyl sulfate (2) | Phenol (3) | 0.22 | 1.44E-07 |
| Cortisol (3) | 18-Hydroxy-11-dehydrotetrahydrocorticosterone (2) | 0.22 | 3.48E-07 |
| Capric acid (2) | Dodecanoic acid (2) | 0.21 | 4.14E-07 |
| Perfluorohexanesulfonic acid (3) | Perfluorooctane sulfonic acid (3) | 0.21 | 5.34E-07 |
| 19-Hydroxyandrost-4-ene-3,17-dione (2) | Cortisone (2) | 0.21 | 6.25E-07 |
| 5a-Dihydrotestosterone sulfate (2) | Androsterone glucuronide (2) | 0.21 | 7.15E-07 |
| Pentadecanoic acid (3) | Heptadecanoic acid (3) | 0.21 | 8.99E-07 |
| Cortisone (2) | 5a-dihydrotestosterone sulfate (2) | 0.20 | 1.45E-06 |
| Perfluorooctanoic acid (3) | Perfluorononanoic acid (3) | 0.20 | 1.53E-06 |
| Stearidonic acid (2) | Alpha-Linolenic acid (3) | 0.20 | 1.95E-06 |
| Lithocholic acid glycine conjugate (3) | Sulfolithocholylglycine (2) | 0.20 | 2.68E-06 |
| Diphenyl phosphate (1) | Coproporphyrin III (3) | 0.20 | 2.81E-06 |
| Perfluorooctyl ethanoic acid (1) | Pentachlorophenol (1) | 0.20 | 2.83E-06 |
| Diphenyl phosphate (1) | 4-Propoxyphenol (3) | 0.19 | 4.76E-06 |
| Stearidonic acid (2) | Eicosapentaenoic acid (2) | 0.19 | 5.62E-06 |
| cis-4-Hydroxycyclohexylacetic acid (3) | 9,10,13-TriHOME (2) | 0.19 | 5.89E-06 |
| p-Cresol (3) | Alpha-N-Phenylacetyl-L-glutamine (3) | 0.19 | 6.38E-06 |
| 6-Tetradecenoic acid (2) | Palmitelaidic acid (2) | 0.19 | 6.82E-06 |
| Deoxycholic acid glycine conjugate (2) | Glycocholic acid (3) | 0.19 | 9.59E-06 |
| 17-Hydroxypregnenolone sulfate (2) | Pregnanediol-3-glucuronide (3) | 0.19 | 1.23E-05 |
| 10Z-Heptadecenoic acid (3) | Nonadeca-10Z-enoic acid (3) | 0.18 | 1.49E-05 |
| Mono (2-ethylhexyl) phthalate (1) | Perfluorohexanesulfonic acid (3) | 0.18 | 1.54E-05 |
| Caprylic acid (3) | Dodecanoic acid (2) | 0.18 | 1.82E-05 |
| Dehydroepiandrosterone sulfate (2) | 17-Hydroxypregnenolone sulfate (2) | 0.18 | 2.13E-05 |
| Octylphenol diethoxylate (3) | Alpha-Linolenic acid (3) | 0.18 | 2.20E-05 |
| Capric acid (2) | 6-Tetradecenoic acid (2) | 0.18 | 2.32E-05 |
| Alpha-Linolenic acid (3) | Linoleic acid (3) | 0.18 | 3.33E-05 |
| 2-Methoxyestrone (3) | Cortisone (2) | 0.18 | 3.58E-05 |
| Arachidonic acid (3) | Adrenic acid (3) | 0.17 | 4.95E-05 |
| Cortisone (2) | Calcitriol (3) | 0.17 | 7.75E-05 |
| L-Tryptophan (3) | Arachidonic acid (3) | 0.17 | 8.76E-05 |
| Eicosadienoic acid (2) | Eicosenoic acid (3) | 0.17 | 8.85E-05 |
| LysoPC(16:0) (2) | LysoPC(17:0) (2) | 0.17 | 9.16E-05 |
| Eicosapentaenoic acid (2) | Docosapentaenoic acid (3) | 0.16 | 0.000107 |
| Estragole (3) | Paraxanthine (2) | 0.16 | 0.000109 |
| Nonadeca-10Z-enoic acid (3) | Eicosenoic acid (3) | 0.16 | 0.000111 |
| 11,14,17-Eicosatrienoic acid (3) | Adrenic acid (3) | 0.16 | 0.000117 |
| L-Tryptophan (3) | Myristic acid (2) | 0.16 | 0.000125 |
| Monoundecyl phthalate (3) | Pentachlorophenol (1) | 0.16 | 0.000133 |
| Pregnenolone sulfate (3) | Pregnanediol-3-glucuronide (3) | 0.16 | 0.000135 |
| Prostaglandin J2 (2) | 2-Arachidonylglycerol (3) | 0.16 | 0.000147 |
| Androstanediol (3) | Docosahexaenoic acid (2) | 0.16 | 0.00016 |
| Mono (2-ethylhexyl) phthalate (1) | Monoundecyl phthalate (3) | 0.16 | 0.00016 |
| 9,10,13-TriHOME (2) | Bilirubin (3) | 0.16 | 0.000178 |
| LysoPC(14:0) (2) | APGPR Enterostatin (3) | 0.16 | 0.000196 |
| L-Tryptophan (3) | 9- or 13-HODE (2) | -0.16 | 0.000158 |
| Monoundecyl phthalate (3) | Perfluorooctane sulfonic acid (3) | -0.16 | 0.000149 |
| Ethyl paraben (1) | Linoleic acid (3) | -0.16 | 0.000129 |
| Mono (2-carboxymethylhexyl) phthalate (3) | Paraxanthine (2) | -0.17 | 4.52E-05 |
| Estragole (3) | Ethyl paraben (1) | -0.17 | 4.22E-05 |
| Mono (2-ethylhexyl) phthalate (1) | Monohydroxyisononyl phthalate (3) | -0.19 | 1.26E-05 |
| Calcitriol (3) | PI(16:1(9Z)18:0 (3) | -0.21 | 1.08E-06 |

**Table S3**. **Summary of significant partial correlations between compounds found in women firefighters**

| **Compound 1 (ID level^1^)** | **Compound 2 (ID level^1^)** | **PCC^2^** | ***P* value** |
| --- | --- | --- | --- |
| Caprylic acid (3) | Capric acid (2) | 0.29 | 2.22E-16 |
| 2-(1,1-Dimethylethyl)phenol (3) | Thymol (3) | 0.29 | 2.22E-16 |
| Glycocholic acid (3) | Cholesterol sulfate (3) | 0.26 | 1.47E-14 |
| Taurodeoxycholic acid (2) | Taurocholic acid (3) | 0.25 | 1.38E-13 |
| Dodecanoic acid (2) | 6-Tetradecenoic acid (2) | 0.24 | 1.26E-12 |
| Cresol (2) | Cresol sulfate (2) | 0.24 | 1.99E-12 |
| 2-Methoxyestrone (3) | 19-Hydroxyandrost-4-ene-3,17-dione (2) | 0.22 | 5.44E-11 |
| Lithocholic acid (3) | Sulfolithocholylglycine (2) | 0.21 | 6.04E-10 |
| Cholesterol sulfate (3) | Taurocholic acid (3) | 0.21 | 8.29E-10 |
| 12-KETE (2) | HDoHE (3) | 0.21 | 1.49E-09 |
| Glycocholic acid (3) | Taurocholic acid (3) | 0.20 | 5.04E-09 |
| Capric acid (2) | Dodecanoic acid (2) | 0.20 | 1.08E-08 |
| Androsterone glucuronide (2) | 11-beta-Hydroxyandrosterone-3-glucuronide (2) | 0.19 | 1.50E-08 |
| Indoxyl sulfate (2) | Alpha-N-Phenylacetyl-L-glutamine (3) | 0.19 | 2.53E-08 |
| 5a-dihydrotestosterone sulfate (2) | Androsterone glucuronide (2) | 0.18 | 9.89E-08 |
| Pregnenolone sulfate (3) | 17-Hydroxypregnenolone sulfate (2) | 0.18 | 1.20E-07 |
| 2-Arachidonylglycerol (3) | Chenodeoxycholic acid (3) | 0.18 | 1.65E-07 |
| Eicosapentaenoic acid (2) | Docosahexaenoic acid (2) | 0.18 | 2.01E-07 |
| LysoPC(15:0) (2) | APGPR Enterostatin (3) | 0.18 | 2.03E-07 |
| LysoPC(14:0) (2) | LysoPC(16:0) (2) | 0.18 | 2.52E-07 |
| Lithocholic acid (3) | Lithocholic acid glycine conjugate (3) | 0.17 | 3.83E-07 |
| PFHxS (1) | PFOS (1) | 0.17 | 7.89E-07 |
| Cortisone (2) | Dehydroepiandrosterone sulfate (2) | 0.17 | 1.43E-06 |
| Indoxyl sulfate (2) | Phenol (3) | 0.16 | 1.68E-06 |
| Methyl eugenol (3) | Butyl paraben (1) | 0.16 | 2.28E-06 |
| Caprylic acid (3) | Dodecanoic acid (2) | 0.16 | 2.64E-06 |
| Cortisol (3) | 18-Hydroxy-11-dehydrotetrahydrocorticosterone (2) | 0.16 | 3.54E-06 |
| Monoisoheptyl phthalate (3) | 4-Ethylbenzoic acid (3) | 0.16 | 4.65E-06 |
| Capric acid (2) | 6-Tetradecenoic acid (2) | 0.16 | 5.39E-06 |
| L-Tryptophan (3) | Myristic acid (2) | 0.15 | 6.15E-06 |
| Diphenyl phosphate (10 | 4-Propoxyphenol (3) | 0.15 | 6.35E-06 |
| cis-4-Hydroxycyclohexylacetic acid (3) | 9,10,13-TriHOME (2) | 0.15 | 8.68E-06 |
| Docosahexaenoic acid (2) | Docosapentaenoic acid (3) | 0.15 | 8.74E-06 |
| Monoisotridecyl phthalate (3) | Tetrahydrocorticosterone (3) | 0.15 | 1.06E-05 |
| 4-Hydroxyphenone (2) | Lithocholic acid glycine conjugate (3) | 0.15 | 1.08E-05 |
| Stearidonic acid (2) | Alpha-Linolenic acid (3) | 0.15 | 1.35E-05 |
| Cresol (2) | Alpha-N-Phenylacetyl-L-glutamine (3) | 0.15 | 1.59E-05 |
| 4-Hydroxyphenone (2) | Benzene-1,2-diol (3) | 0.15 | 1.67E-05 |
| 19-Hydroxyandrost-4-ene-3,17-dione (2) | Cortisone (2) | 0.15 | 1.91E-05 |
| Nonadeca-10Z-enoic acid (3) | Eicosenoic acid (2) | 0.14 | 2.69E-05 |
| Pregnenolone sulfate (3) | Pregnanediol-3-glucuronide (3) | 0.14 | 2.86E-05 |
| Myristic acid (2) | Pentadecanoic acid (3) | 0.14 | 2.88E-05 |
| 9- or 13-HODE (2) | 9,10-DiHODE (2) | 0.14 | 3.66E-05 |
| PFOS (1) | Calcitriol (3) | 0.14 | 4.43E-05 |
| Diphenyl phosphate (1) | Monoisobutyl phthalate (2) | 0.14 | 4.53E-05 |
| 4-Ethylbenzoic acid (3) | 11b-Hydroxyprogesterone (2) | 0.14 | 4.89E-05 |
| 6-Tetradecenoic acid (2) | Palmitelaidic acid (2) | 0.14 | 5.09E-05 |
| Androstanediol (3) | Docosahexaenoic acid (2) | 0.14 | 5.54E-05 |
| LysoPC(14:0) (2) | APGPR Enterostatin (3) | 0.14 | 5.90E-05 |
| Mono (2-ethylhexyl) phthalate (1) | 12,13-DHOME (2) | 0.14 | 6.10E-05 |
| Monopropyl phthalate (3) | Monoisobutyl phthalate (2) | 0.14 | 6.26E-05 |
| Lithocholic acid glycine conjugate (3) | Sulfolithocholylglycine (2) | 0.14 | 6.51E-05 |
| Pentadecanoic acid (3) | Heptadecanoic acid (3) | 0.14 | 7.42E-05 |
| Monopropyl phthalate (3) | Ethyl paraben (1) | 0.13 | 8.70E-05 |
| Butyl paraben (1) | 3,5-Di-tert-Butylsalicylic acid (3) | 0.13 | 9.94E-05 |
| Arachidonic acid (3) | 15(S)-HETE (2) | 0.13 | 0.000106 |
| Phytanic acid (3) | LysoPC(16:0) (2) | 0.13 | 0.000118 |
| Eicosatrienoic acid (3) | Adrenic acid (3) | 0.13 | 0.000126 |
| Deoxycholic acid glycine conjugate (2) | Glycocholic acid (3) | 0.13 | 0.00013 |
| Monoisobutyl phthalate (2) | Mono (2-carboxymethylhexyl) phthalate (2) | 0.13 | 0.00014 |
| 3,5-Dihydroxybenzoic acid (2) | Paraxanthine (2) | 0.13 | 0.000141 |
| Deoxycholic acid glycine conjugate (2) | Cholesterol sulfate (3) | 0.13 | 0.000151 |
| Benzene-1,2-diol (3) | 4-Ethylbenzoic acid (3) | 0.13 | 0.000156 |
| Pyrethrin II (3) | 9,10,13-TriHOME (2) | 0.13 | 0.000166 |
| 15(S)-HETE (2) | HDoHE (3) | 0.13 | 0.000171 |
| Diphenyl phosphate (1) | Pentachlorophenol (1) | 0.13 | 0.000179 |
| Ethyl paraben (1) | Benzene-1,2-diol (3) | 0.13 | 0.000198 |
| 19-Hydroxyandrost-4-ene-3,17-dione (2) | 11b-Hydroxyprogesterone (2) | 0.13 | 0.000224 |
| Alpha-Linolenic acid (3) | Linoleic acid (3) | 0.13 | 0.00024 |
| Monoisotridecyl phthalate (3) | Myristic acid (2) | 0.13 | 0.000241 |
| PFHxS (1) | Sulfolithocholylglycine (2) | 0.13 | 0.000259 |
| Cortisone (2) | 5a-dihydrotestosterone sulfate (2) | 0.12 | 0.000282 |
| cis-4-Hydroxycyclohexylacetic acid (3) | Paraxanthine (2) | 0.12 | 0.000289 |
| 2,4-Dinitrophenol (3) | PFOA (1) | 0.12 | 0.000306 |
| Eugenol (1) | Bilirubin (3) | 0.12 | 0.000325 |
| Tetranor 12-HETE (3) | Chlorogenic acid (3) | 0.12 | 0.00033 |
| Ethyl paraben (1) | 11-beta-Hydroxyandrosterone-3-glucuronide (2) | -0.12 | 0.000325 |
| PFOS (1) | 15d PGD2 (2) | -0.13 | 0.000247 |
| Monopropyl phthalate (3) | PFOA (1) | -0.13 | 0.000236 |
| Monoundecyl phthalate (3) | Ethyl paraben (1) | -0.13 | 0.000136 |
| 4-Hydroxyphenone (2) | Chenodeoxycholic acid (3) | -0.13 | 0.000101 |
| Monopropyl phthalate (3) | Thymol (3) | -0.14 | 5.92E-05 |
| Monohydroxyisononyl phthalate (3) | 11b-Hydroxyprogesterone (2) | -0.14 | 5.69E-05 |
| L-Tryptophan (3) | 9- or 13-HODE (2) | -0.15 | 1.17E-05 |
| Calcitriol (3) | PI(16:1(9Z)/18:0) (3) | -0.18 | 1.06E-07 |

**Table S4**. **Summary of significant partial correlations between compounds found in women office workers**

| **Compound 1 (MSI level^1^)** | **Compound 2 (MSI level^1^)** | **PCC^2^** | ***P* value** |
| --- | --- | --- | --- |
| 2-(1,1-Dimethylethyl)phenol (3) | Thymol (3) | 0.30 | 1.11E-15 |
| Caprylic acid (3) | Capric acid (2) | 0.27 | 2.43E-13 |
| Myristic acid (2) | 12(13)Ep-9-KODE (2) | 0.26 | 3.59E-12 |
| Cresol (2) | p-Cresol sulfate (2) | 0.26 | 4.52E-12 |
| Perfluorooctanoic acid (3) | Perfluorononanoic acid (3) | 0.25 | 2.96E-11 |
| 2-Arachidonylglycerol (3) | Chenodeoxycholic acid (3) | 0.24 | 4.78E-11 |
| Dodecanoic acid (2) | 6-Tetradecenoic acid (2) | 0.21 | 8.52E-09 |
| Glycocholic acid (3) | Cholesterol sulfate (3) | 0.21 | 1.05E-08 |
| Androsterone glucuronide (2) | 11-beta-Hydroxyandrosterone-3-glucuronide (2) | 0.21 | 3.21E-08 |
| 15(S)-HETE (2) | 19(20)-EpDPE (2) | 0.20 | 1.15E-07 |
| Lithocholic acid (3) | Lithocholic acid glycine conjugate (3) | 0.19 | 2.35E-07 |
| Caprylic acid (3) | Dodecanoic acid (2) | 0.19 | 4.52E-07 |
| 12-KETE (2) | 19(20)-EpDPE (2) | 0.19 | 4.93E-07 |
| Capric acid (2) | Dodecanoic acid (2) | 0.19 | 5.34E-07 |
| Cholesterol sulfate (3) | Taurocholic acid (3) | 0.18 | 8.68E-07 |
| Pentadecanoic acid (3) | Heptadecanoic acid (3) | 0.18 | 1.11E-06 |
| Lithocholic acid (3) | Sulfolithocholylglycine (2) | 0.18 | 2.29E-06 |
| Octylphenol diethoxylate (3) | Alpha-Linolenic acid (3) | 0.18 | 2.74E-06 |
| Diphenyl phosphate (1) | Monoundecyl phthalate (3) | 0.17 | 3.49E-06 |
| Glycocholic acid (3) | Taurocholic acid (3) | 0.17 | 3.57E-06 |
| LysoPC(15:0) (2) | APGPR Enterostatin (3) | 0.17 | 4.21E-06 |
| 2-Methoxyestrone (3) | 19-Hydroxyandrost-4-ene-3,17-dione (2) | 0.17 | 5.24E-06 |
| Indoxyl sulfate (2) | Alpha-N-Phenylacetyl-L-glutamine (3) | 0.17 | 5.48E-06 |
| Deoxycholic acid glycine conjugate (2) | Glycocholic acid (3) | 0.17 | 6.65E-06 |
| Arachidonic acid (3) | 15(S)-HETE (2) | 0.17 | 7.09E-06 |
| 19-Hydroxyandrost-4-ene-3,17-dione (2) | Cortisone (2) | 0.17 | 9.22E-06 |
| Taurochenodesoxycholic acid (3) | Taurocholic acid (3) | 0.17 | 1.05E-05 |
| Stearidonic acid (2) | Eicosapentaenoic acid (2) | 0.16 | 1.18E-05 |
| 2-Methoxyestrone (3) | Cortisone (2) | 0.16 | 1.65E-05 |
| 17-Hydroxypregnenolone sulfate (2) | Androsterone glucuronide (2) | 0.16 | 1.78E-05 |
| Pregnenolone sulfate (3) | 17-Hydroxypregnenolone sulfate (2) | 0.16 | 1.85E-05 |
| L-Tryptophan (3) | Phytanic acid (3) | 0.16 | 1.99E-05 |
| Methyl eugenol (3) | Butyl paraben (1) | 0.16 | 2.51E-05 |
| Cortisol (3) | 18-Hydroxy-11-dehydrotetrahydrocorticosterone (2) | 0.16 | 3.36E-05 |
| Capric acid (2) | 6-Tetradecenoic acid (2) | 0.15 | 3.87E-05 |
| Monoisotridecyl phthalate (3) | 5a-Tetrahydrocorticosterone (3) | 0.15 | 4.18E-05 |
| Eicosapentaenoic acid (2) | Docosahexaenoic acid (2) | 0.15 | 4.46E-05 |
| 12-KETE (2) | 15(S)-HETE (2) | 0.15 | 5.07E-05 |
| Pyrethrin II (3) | Leucine (3) | 0.15 | 5.89E-05 |
| 3,5-Dihydroxybenzoic acid (2) | Palmitelaidic acid (2) | 0.15 | 6.48E-05 |
| Mono (2-ethylhexyl) phthalate (1) | Perfluorohexanesulfonic acid (1) | 0.15 | 6.98E-05 |
| Dehydroepiandrosterone sulfate (2) | Androsterone glucuronide (2) | 0.15 | 7.54E-05 |
| Dehydroepiandrosterone sulfate (2) | 17-Hydroxypregnenolone sulfate (2) | 0.15 | 9.33E-05 |
| Pentachlorophenol (1) | 12S-HHT (2) | 0.15 | 9.80E-05 |
| Alpha-Linolenic acid (3) | 9- or 13-HODE (2) | 0.15 | 0.000102 |
| Monoisobutyl phthalate (2) | Perfluorooctanoic acid (3) | 0.15 | 0.000113 |
| Cresol (2) | Alpha-N-Phenylacetyl-L-glutamine (3) | 0.14 | 0.000117 |
| Perfluorohexanesulfonic acid (1) | 4-Propoxyphenol (3) | 0.14 | 0.00012 |
| Perfluorooctyl ethanoic acid (1) | Pentachlorophenol (1) | 0.14 | 0.000136 |
| Pentachlorophenol (1) | Calcitriol (3) | -0.14 | 0.000131 |
| Monoisobutyl phthalate (2) | 9,10-DiHODE (2) | -0.14 | 0.000121 |
| Monoisobutyl phthalate (2) | 9- or 13-HODE (2) | -0.15 | 6.05E-05 |
| Butyl paraben (1) | Eicosapentaenoic acid (2) | -0.15 | 4.55E-05 |
| Mono (2-ethylhexyl) phthalate (1) | Monohydroxyisononyl phthalate (3) | -0.17 | 7.14E-06 |

^1^Metabolomics Standard Initiative (MSI) level of identification; level 1: match based on accurate mass (± 10 ppm), fragmentation pattern and relative retention time with authentic standards; level 2: match based on accurate mass (± 10 ppm) and fragmentation pattern using mass spectra from public metabolomics libraries or in-silico fragmentation software; level 3: match based on accurate mass (± 10 ppm) only. ^2^Partial Correlation Coefficient (PCC)

**References**

1. Uppal, K., Walker, D. I. & Jones, D. P. xMSannotator: An R Package for Network-Based Annotation of High-Resolution Metabolomics Data. *Anal. Chem.* **89**, 1063–1067 (2017).

2. Smith, C. A., Want, E. J., O’Maille, G., Abagyan, R. & Siuzdak, G. XCMS:  Processing Mass Spectrometry Data for Metabolite Profiling Using Nonlinear Peak Alignment, Matching, and Identification. *Anal. Chem.* **78**, 779–787 (2006).

3. Schaefer, J., Opgen-Rhein, R. & Strimmer, K. *GeneNet: Modeling and Inferring Gene Networks*. (2020).

4. Calafat, A. M., Wong, L.-Y., Kuklenyik, Z., Reidy, J. A. & Needham, L. L. Polyfluoroalkyl chemicals in the U.S. population: data from the National Health and Nutrition Examination Survey (NHANES) 2003-2004 and comparisons with NHANES 1999-2000. *Environ. Health Perspect.* **115**, 1596–1602 (2007).

5. Calafat, A. M., Ye, X., Wong, L.-Y., Bishop, A. M. & Needham, L. L. Urinary Concentrations of Four Parabens in the U.S. Population: NHANES 2005–2006. *Environ. Health Perspect.* **118**, 679–685 (2010).

6. CDC. CDC. National Health and Nutrition Examination Survey: Anthropometry Procedures Manual. https://www.cdc.gov/nchs/data/nhanes/nhanes_07_08/manual_an.pdf (2009).

7. CDC. CDC. National Health and Nutrition Examination Survey: Laboratory Procedures Manual. https://www.cdc.gov/nchs/data/nhanes/nhanes_09_10/lab.pdf (2009).

8. CDC. CDC. Laboratory Procedure manual. C-Reactive Protein. https://wwwn.cdc.gov/nchs/data/nhanes/2009-2010/labmethods/CRP_F_met.pdf (2011).

9. CDC. CDC, National Center for Health Statistics . National Health and Nutrition Examination Survey. Dietary Supplements and Prescription Medication-DSQ. (2012).

10. Multum Lexicon database. National Health and Nutrition Examination Survey: 1988–2012 Data Documentation, Codebook, and Frequencies. (2009).

11. Sumner, L. W. *et al.* Proposed minimum reporting standards for chemical analysis Chemical Analysis Working Group (CAWG) Metabolomics Standards Initiative (MSI). *Metabolomics Off. J. Metabolomic Soc.* **3**, 211–221 (2007).
